# Adjunctive selective estrogen receptor modulator increases neural activity in the hippocampus and inferior frontal gyrus during emotional face recognition in schizophrenia

**DOI:** 10.1038/tp.2016.59

**Published:** 2016-05-03

**Authors:** E Ji, C S Weickert, R Lenroot, J Kindler, A J Skilleter, A Vercammen, C White, R E Gur, T W Weickert

**Affiliations:** 1 School of Psychiatry, University of New South Wales, Randwick, NSW, Australia; 2 Neuroscience Research Australia, Randwick, NSW, Australia; 3 Schizophrenia Research Institute, Darlinghurst, NSW, Australia; 4 University Hospital of Child and Adolescent Psychiatry, University of Bern, Bern, Switzerland; 5 Department of Endocrinology, Prince of Wales Hospital, Randwick, NSW, Australia; 6 Department of Psychiatry, Perelman School of Medicine, University of Pennsylvania, Philadelphia, PA, USA

## Abstract

Estrogen has been implicated in the development and course of schizophrenia with most evidence suggesting a neuroprotective effect. Treatment with raloxifene, a selective estrogen receptor modulator, can reduce symptom severity, improve cognition and normalize brain activity during learning in schizophrenia. People with schizophrenia are especially impaired in the identification of negative facial emotions. The present study was designed to determine the extent to which adjunctive raloxifene treatment would alter abnormal neural activity during angry facial emotion recognition in schizophrenia. Twenty people with schizophrenia (12 men, 8 women) participated in a 13-week, randomized, double-blind, placebo-controlled, crossover trial of adjunctive raloxifene treatment (120 mg per day orally) and performed a facial emotion recognition task during functional magnetic resonance imaging after each treatment phase. Two-sample *t*-tests in regions of interest selected *a priori* were performed to assess activation differences between raloxifene and placebo conditions during the recognition of angry faces. Adjunctive raloxifene significantly increased activation in the right hippocampus and left inferior frontal gyrus compared with the placebo condition (family-wise error, *P<*0.05). There was no significant difference in performance accuracy or reaction time between active and placebo conditions. To the best of our knowledge, this study provides the first evidence suggesting that adjunctive raloxifene treatment changes neural activity in brain regions associated with facial emotion recognition in schizophrenia. These findings support the hypothesis that estrogen plays a modifying role in schizophrenia and shows that adjunctive raloxifene treatment may reverse abnormal neural activity during facial emotion recognition, which is relevant to impaired social functioning in men and women with schizophrenia.

## Introduction

Schizophrenia is a disabling psychiatric disorder, with a 70–80% unemployment rate,^1^ associated with multifaceted deficits in cognitive function^2, 3^ and emotion processing.^4, 5^ Although antipsychotics are the first line of treatment for schizophrenia, these medications are often limited in their effectiveness and leave many patients with residual symptoms while producing unwanted side effects.^6^ There is growing evidence that sex hormones may influence the course and symptoms of schizophrenia. The onset of the disease typically occurs during adolescence^7^ and the clinical presentation, response to treatment and symptom severity can differ between men and women.^8, 9, 10^ Schizophrenia occurs less frequently and has a later average age of onset in women.^11, 12^ Furthermore, women tend to experience a less-severe course of the disease compared with men.^9^ Studies have found lower estrogen levels in women with schizophrenia relative to healthy women, relapses are more frequent when estrogen levels are low, such as during the early follicular phase of the menstrual cycle, postpartum and after menopause when there is a second peak of illness onset and a more severe course of illness.^13, 14, 15, 16, 17, 18^ These findings support the estrogen hypothesis of schizophrenia that posits that estrogen may have a neuroprotective effect against the disease.^19^

Alterations in dopaminergic and serotonergic systems are also key components of schizophrenia pathogenesis and animal research has shown a modulatory effect of estrogen upon dopamine and serotonin neurotransmitter systems in the brain.^20, 21, 22, 23^ The neurobiological benefit of estrogen^24^ has led to an increase in the number of studies investigating estrogen as a potential therapeutic treatment in schizophrenia.^25, 26, 27, 28, 29, 30^ Despite growing positive evidence that estrogen may reduce symptom severity in schizophrenia and may benefit cognition during aging,^31, 32, 33^ little is known regarding the therapeutic effects of estrogen on aspects of schizophrenia that are relatively unresponsive to standard therapeutic intervention, such as cognitive dysfunction and social impairment.

Selective estrogen receptor modulators (SERMs) specifically activate estrogen receptors and no other nuclear receptors.^34, 35^ Unlike estrogen, SERMs do not stimulate estrogen receptors in the breast or uterine tissue (therefore, avoiding any adverse effects in these tissues). Raloxifene is a first-generation SERM that is used to treat osteoporosis in postmenopausal women^36^ and has demonstrated benefits in preventing age-related decreases in neural activity in healthy older men.^37^ More recently, adjunctive treatment with raloxifene in postmenopausal women with schizophrenia has demonstrated reductions in positive and negative symptom severity and general psychopathology.^38, 39^ A recent clinical trial has shown that adjunctive raloxifene administered at 60 mg per day improved memory and verbal fluency in postmenopausal women with schizophrenia.^40^ In addition, we have shown that daily, oral adjunctive raloxifene treatment at 120 mg per day improved verbal memory and attention and increased the brain activity during learning in both men and women with schizophrenia.^41, 42^ However, no clinical trial to date has examined the extent to which adjunctive raloxifene treatment may influence facial emotion recognition, which is a critical skill linked to social function in men and women with schizophrenia.^43^

Poor social functioning is a core feature of schizophrenia^44^ that is associated with deficits in emotion processing in which people with schizophrenia often have difficulty identifying and discriminating among different facial expressions.^45^ Most evidence suggests that people with schizophrenia display hypoactivation in frontal and limbic regions and perform worse relative to healthy individuals during facial emotion identification tasks.^46, 47, 48^ The regions of particular interest in relation to facial emotion recognition are the bilateral amygdala, hippocampus and inferior frontal gyrus (IFG). Amygdala damage has been linked to impaired recognition of fearful and angry facial expressions.^49, 50, 51^ Further, amygdala dysfunction has been implicated in facial emotion processing tasks in people with schizophrenia, with several studies reporting both hyper- or hypoactivation.^52, 53, 54, 55^ Thus, although functional abnormality of the amygdala in schizophrenia is well established, the exact direction of the disruption remains unclear.

The neural activity in the hippocampus is associated with amygdala activity and emotional memory. The hippocampus represents a key interface between sensory systems and the limbic system, and is necessary to form and elicit long-term memories.^56^ It has been suggested that the hippocampus encodes the emotional sense of experiences so that they may be recalled at a later time in association with an emotional valence.^57^ Together, the hippocampus and amygdala interact during emotion processing and emotional memory retrieval.^58, 59^

Regarding the cortical IFG, a meta-analysis of 105 functional magnetic resonance imaging (fMRI) studies in healthy participants reported significantly increased IFG activation during the processing of angry faces relative to a baseline control condition.^60^ In schizophrenia, the fMRI studies report abnormal IFG activation during semantic processing^61^ and emotion processing.^62^ We recently reported lower levels of neural activity in the left IFG in schizophrenia relative to healthy controls during recognition of negative facial emotions.^47^

The aim of the present study was to determine the extent to which a hormone intervention therapy using the SERM raloxifene will influence neural activity underlying recognition of facial emotions in men and women with schizophrenia. For the fMRI analysis, we focused on cortical and subcortical regions of interest where there is evidence of functional abnormalities during the facial emotion recognition in people with schizophrenia compared with healthy controls: the amygdala, hippocampus and IFG. We focused on angry versus neutral faces because people with schizophrenia are particularly impaired in the identification of faces displaying negative emotions.^63, 64^ Further, there is evidence of a relationship between the magnitude of brain activity during processing of direct social threat (anger) and symptom improvement in people with schizophrenia, whereas this relationship was not found for indirect threat (fear).^65^ We predicted that administration of raloxifene will increase brain activity in the hippocampus and IFG in men and women with schizophrenia, compared with the placebo. Owing to contrasting reports regarding the direction of abnormal amygdala activation in schizophrenia, we had a non-directional hypothesis that raloxifene would have a significant influence on neural activity relative to placebo in the amygdala.

## Materials and methods

### Participants

The study sample consisted of 20 people with schizophrenia or schizoaffective disorder (12 male, 8 female). Patients were recruited via a national television documentary, the outpatient mental health unit at the Prince of Wales Hospital and community mental health clinics in the South Eastern Sydney and Illawarra Area Health Service. All the patients were between 22 and 51 years of age and were receiving antipsychotic medication for at least 1 year before taking part in the study. Clinical diagnostic interviews using the Structured Clinical Interview for DSM-IV^66^ were performed by a trained psychologist or psychiatrist. The symptom severity was assessed using the Positive and Negative Syndrome Scale (PANSS).^67^ The duration of illness was defined as the difference between the age at first hospitalization and age at the time of scanning. The exclusion criteria included a comorbid Axis I DSM-IV disorder, substance abuse or dependency within the past 5 years, seizures, central nervous system infection, uncontrolled diabetes or hypertension, a history of neurological illness, head injury with loss of consciousness, structural brain abnormalities as assessed by MRI scan, intellectual disability (current intelligence quotient <70) or contraindications to the administration of raloxifene. Women were excluded if they were currently pregnant or were receiving hormone therapy and refused alternate forms of birth control. See Table 1 for demographic and clinical characteristics of the sample.

The fasting peripheral blood samples were collected between 0900 and 1100 h to control for alterations in hormone levels due to diurnal variations. The clotted and heparinized blood were delivered on ice to the Prince of Wales Hospital South Eastern Area Laboratory Services Pathology Unit immediately following collection. Prolactin, follicle-stimulating hormone and luteinizing hormone were assayed using a chemiluminescent immunometric assay (Siemens Immulite 2000, Bayswater, VIC, Australia).

The participants were assessed with the Wechsler Test of Adult Reading^68^ to obtain an estimate of premorbid intellectual functioning, and a four subtest version of the Wechsler Adult Intelligence scale, third edition^69^ comprising the Arithmetic, Digit Symbol, Similarities and Picture Completion subtests to assess current intellectual functioning. The study procedures were approved by the University of New South Wales and the South Eastern Sydney and Illawarra Area Health Service Ethic Committees. All the participants provided written informed consent before participation in the study.

### Study design

In a 13-week, randomized, double-blind, placebo-controlled, crossover trial patients received 120 mg per day of encapsulated raloxifene and placebo (encapsulated lactose) as an adjunctive treatment to their currently prescribed antipsychotic medication (see Supplementary Figure 1). All quality assessment/control testing of encapsulated raloxifene was performed by IDT Australia (Boronia, VIC, Australia). Following a baseline assessment, the participants were randomly assigned to receive raloxifene (10 patients) or placebo (10 patients) for 6 weeks using a computer-generated randomization schedule provided by the Prince of Wales Hospital Pharmacy Clinical Trials Unit. Following the first 6-week period of the trial, there was a 1 week ‘washout' period followed by the second 6-week period when patients received the alternate treatment (raloxifene or placebo). The patients were monitored throughout the trial to assess potential adverse events and the compliance was determined by returned pill counts and hormonal blood assays. The fMRI was used to measure blood oxygen level-dependent (BOLD) signal changes as the patients performed a facial emotion recognition task at week 6 (end of trial period 1) and at week 13 (end of trial period 2).

### Facial emotion recognition task

During each test session, the participants completed a facial emotion recognition task in the scanning environment (see Supplementary Figure 2). The stimuli consisted of 60 unique color pictures of human faces representing equal numbers of the following emotions: anger, fear, happy, sad and neutral.^46^ Each of the five emotional expressions was presented a total of 12 times. The stimuli were presented on an inverted computer screen via a set of mirrors in the scanner for 5.5 s each and for each presentation, the individuals were asked to identify the affect displayed using a button response box.

### Demographic and task statistical analyses

The data analysis was performed using IBM SPSS 22 for Windows (Armonk, NY, USA). Antipsychotic dose, hormone levels, PANSS scores and task performance measures were compared between treatment conditions using *t*-tests or Wilcoxon rank-sum tests as appropriate. Task performance included measures of accuracy (% correct responses) and reaction times for each facial expression. Owing to technical problems, the behavioral data were limited to 17 patients during the active raloxifene phase and 11 patients during the placebo phase.

### Image acquisition and processing

Echoplanar MR brain images were acquired using a 3-Tesla Phillips Achieva MRI scanner with an eight-channel bird-cage-type head coil at the Neuroscience Research Australia, Randwick, NSW, Australia. Each participant received a T1-weighted high-resolution anatomical scan to screen for structural abnormalities and for co-registration (TR (repetition time): 5.4 ms; TE (echo time): 2.4 ms; field of view: 256 mm; matrix: 256 × 256; sagittal plane; slice thickness: 1 mm, no gap; 180 slices). During the facial emotion recognition task, 210 T2*-weighted MR images providing BOLD contrast (TR/TE=3000/30; 21 interleaved slices, slice thickness=3.0 mm, gap=1.0 mm, voxel size=3 × 3 × 3 mm; flip angle=90° field of view=24 cm) were acquired. Three dummy scans were obtained before each fMRI data acquisition to allow for the equilibration of the MRI signal.

The BOLD fMRI data were preprocessed and analyzed using Statistical Parametric Mapping software (SPM8; http://www.fil.ion.ucl.ac.uk/spm) running under MATLAB version 2012b. For each participant, the 210 volume functional time series images were realigned to the first image in the sequence and coregistered to the T1 anatomical scan. The images were transformed into stereotactic space (Montreal Neurological Institute) and smoothed with a 10 mm full-width half maximum Gaussian filter. All data sets were screened for artefacts, excessive movement exceeding 3 mm translation on *x*, *y* or *z* axes and successful normalization. The motion parameters were included as regressors in the first-level analysis to further control for motion effects.

### fMRI analyses

At the first level of analysis, a contrast was created for subject-level time series to assess the difference in BOLD signal between conditions of interest: angry faces versus neutral faces. At the second level, we first constructed single-sample *t*-test models for the active and placebo conditions separately at the whole-brain level to assess the main task effect for the recognition of angry faces. Next, to assess activation differences between raloxifene and placebo conditions in specific regions selected *a priori*, which have been previously shown to have a lower level of activation during emotional face recognition in schizophrenia,^46, 47, 48^ we performed paired *t*-tests between raloxifene and placebo conditions in the regions of interest (ROIs). The following bilateral structural ROIs were selected from the Anatomical Automatic Labelling Atlas in the SPM8 toolbox:^70^ amygdala, hippocampus and IFG. We corrected for multiple comparisons across the whole-brain one-sample *t*-tests using false discovery rate (FDR) corrections (*P<*0.05). Small volume corrections were applied for ROIs (family-wise error, *P*<0.05).

## Results

### Demographics, symptom measures, compliance and blood analyses

Demographic and clinical characteristics of the study sample are presented in Table 1. The men and women with schizophrenia in this sample were chronically ill, treated primarily with second-generation antipsychotics, and displayed mild-to-moderate symptom severity based on PANSS scores. On the basis of returned pill counts, compliance for period 1 of the trial was 97.6% and 97.3% for period 2 for this fMRI study. There were no severe adverse events that were attributed to the study medication. There was no statistically significant difference between PANSS-positive and -negative symptoms scores during raloxifene and placebo conditions; however, there was a trend towards a statistically significant decrease in PANSS-positive symptom-severity scores with raloxifene treatment. There were no clinically relevant differences on the hormone panel measures between the raloxifene and placebo conditions although there was a statistically significant increase in follicle-stimulating hormone levels with raloxifene treatment.

### Behavioral results

The performance measures of participants on the facial emotion recognition task included reaction times and response accuracy (% correct) presented in Supplementary Table 1. The Wilcoxon signed-rank tests indicated that there was no significant difference in accuracy and reaction time between adjunctive raloxifene and placebo conditions.

### Imaging

**Figure 1:**
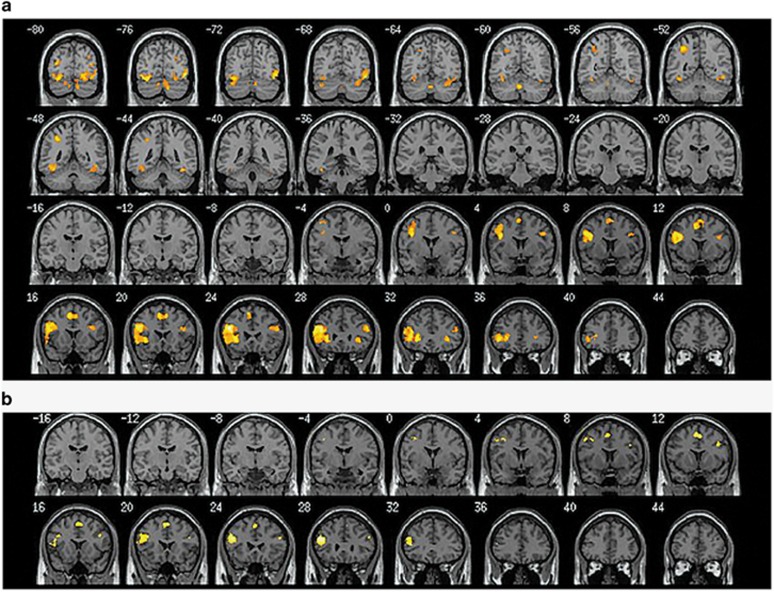
Coronal slices depicting areas of significant neural activation during angry facial recognition in the (**a**) raloxifene and (**b**) placebo conditions.

Whole-brain one-sample *t*-tests for angry versus neutral face recognition revealed significant activation for the raloxifene and placebo conditions (see Figure 1, Table 2). During raloxifene treatment, angry face recognition elicited bilateral activation in a widespread network including inferior, middle, medial and superior frontal gyrus, middle temporal gyrus, inferior parietal lobe, insula, lingual gyrus, fusiform gyrus, parahippocampal gyrus and cingulate gyrus (FDR, *P<*0.05). The placebo condition elicited activation in a similar, yet more restricted network including inferior, middle, medial and superior frontal gyrus and precentral gyrus (FDR, *P*<0.05).

**Figure 2:**
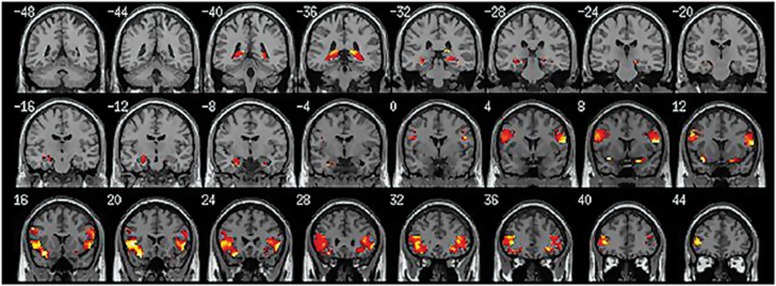
Patients showed significantly greater activation within the left inferior frontal gyrus and right hippocampus (small volume family-wise error corrected, *P*<0.05.) during raloxifene treatment relative to the placebo condition.

When comparing the three brain regions selected *a priori* by ROI (amygdala, hippocampus and IFG bilaterally), the patients showed greater activation only within the bilateral inferior frontal gyrus and hippocampus with raloxifene treatment relative to the placebo condition; however, only the left inferior frontal gyrus and right hippocampus reached the strict statistical significance level (see Figure 2, Table 3). The placebo condition did not elicit greater activation than the raloxifene treatment in any region during recognition of angry faces. There were no significant activation differences between raloxifene and placebo conditions in relation to the amygdala ROI.

## Discussion

The main aim of the present study was to determine the extent to which raloxifene would influence neural activity associated with recognition of faces with a negative valence in men and women with schizophrenia. In accordance with our hypothesis, angry face recognition elicited significantly greater activation in the left IFG and right hippocampus during raloxifene treatment relative to placebo of the same individuals with schizophrenia studied over time. However, we did not detect any change in performance accuracy over the course of the study with raloxifene administration during facial emotion recognition in people with schizophrenia.

The hippocampus is involved in emotion processing and has been shown to be hypoactive in schizophrenia during facial emotion processing tasks. Using the same emotion-recognition paradigm as in the present study, Gur *et al.*^46^ found that people with schizophrenia showed significantly less activation in the bilateral hippocampus relative to healthy controls during exposure to positive and negative facial expressions. Although we did not find significant increases in hippocampal activity in both the hemispheres, we were able to detect an increase in activity of the right hippocampus. Our finding of increased activation in ROIs that are hemisphere-specific supports previous work showing evidence for the laterization of emotion processing.^71^ Papanicolaou *et al.*^72^ demonstrated specialization of the hippocampus in healthy individuals where the left hippocampus activates during mnemonic processing of verbal items and the right side activates for processing visual stimuli that are difficult to encode verbally. Similarly, Bellace *et al.*^57^ reported greater activation in the left hippocampus for emotional words and the right hippocampus for emotional pictures. Another fMRI study reported that encoding faces was associated primarily with activation in the right hippocampus.^73^ Thus, our finding of significantly greater activation in the right hippocampus during raloxifene compared with placebo may reflect the ability of raloxifene to enhance neural processing of emotional visuospatial stimuli.

Several fMRI studies of emotion processing in schizophrenia have found abnormal activity in cortical regions, particularly the IFG. We previously reported hypoactivation of the left IFG in people with schizophrenia relative to healthy controls during angry face processing where matching the emotional face to the correct emotional word is required.^47^ Similarly, Russell *et al.*^74^ showed less BOLD signal in the left IFG during a socioemotional task in men with schizophrenia compared with healthy controls. Furthermore, decreased functional connectivity of the IFG, which may contribute to abnormalities during emotion processing, has been reported during semantic processing^61^ and cognitive processing.^75^ Goekoop *et al.*^37^ reported increased activation in the left IFG following raloxifene treatment compared with placebo in healthy elderly males during a face recognition task. Our finding of increased activation in the left IFG with adjunctive raloxifene supports findings of Goekoop *et al.*^37^ on the effect of raloxifene on brain activity. Further, the left IFG includes Broca's area which has a role in verbal processing.^76^ The paradigm in the present study requires subjects to match the facial emotion with the written emotion displayed, thus marked increase in activation in the left IFG during raloxifene may reflect increased verbal processing to identify the presented visuospatial stimuli.

We did not observe any significant changes in brain activation in the amygdala during recognition of angry faces between raloxifene and placebo conditions. Some neuroimaging studies report hypoactivation of the amygdala during processing of facial emotions, while others report normal or enhanced activation of this region in people with schizophrenia.^52, 53, 54, 55^ One study sought to determine whether variability in amygdala activation may be related to the time course of the experiment. People with schizophrenia initially showed hyper-responsivity of the amygdala to facial expressions relative to healthy controls; whereas in the latter phase of the experiment, patients exhibited decreased bilateral amygdala response.^77^ It has been well established that amygdala response to facial expressions habituates with repeated presentations;^78, 79^ however, Suslow *et al.*^77^ demonstrated a more rapid decrease in amygdala response over time in schizophrenia relative to healthy people. Therefore, heterogeneous findings regarding amygdala activation during emotional face processing may result from differences in length of the experimental paradigms, such that longer tasks are associated with habituation of amygdala activity and therefore less activation when averaged across time. Our study did not show a significant difference in the level of amygdala activation during placebo and raloxifene conditions in schizophrenia. It is possible that raloxifene does not affect activation in this region or that any increase in activation was limited to the initial stimuli presentations and thus, was not significant when we averaged across the whole task.

The exact mechanism by which raloxifene exerts its effects is not entirely known, but may involve neurotransmitter systems or other neuromodulators. The action of raloxifene on mood may be related to mimicking estrogenic effects on pre- and postsynaptic modulation of serotonergic and/or dopaminergic neurotransmission.^80, 81, 82^ A large body of evidence has shown that dopamine neurotransmission influences hippocampal plasticity and function,^83, 84, 85^ and there is an increase in dopaminergic activity in the left inferior frontal gyrus during human emotion processing.^86^ Raloxifene has also been shown to decrease the inflammatory response in mouse and rat microglia cells *in vitro*.^87^ Our group recently found increased inflammatory mRNA expression in a subgroup of people diagnosed with schizophrenia (approximately 40%) suggesting that anti-inflammatory therapies may be of benefit.^88, 89^ Thus, it is possible that the ability of raloxifene to reverse abnormalities in brain activation in patients may, in part, be due to its ability to suppress inflammation. Further, a number of studies have shown that raloxifene has antioxidant properties;^90, 91^ therefore, its beneficial effects in schizophrenia may be related to the control of oxidative stress, which has been shown to be elevated in people with schizophrenia.^92^

The hippocampus is one of the primary sites of estrogen receptors in the brain; and in animal studies, estrogen administration has been linked to increases in synaptic spine density.^93^ Raloxifene has also been shown to reduce neuronal loss in the hippocampus.^94^ Our group previously demonstrated increased activation of the right hippocampus during probabilistic association learning with adjunctive raloxifene.^41^ Thus, our present finding of increased neural activation in the hippocampus during raloxifene administration is consistent with these previous findings of the role of estrogen receptor modulation in the hippocampus. We also find increased activity in the prefrontal cortex of people with schizophrenia, a brain region also expressing estrogen receptors.^95, 96^ Thus, the neural substrate of SERM action may include direct effects in both the hippocampus and IFG.

There are a number of limitations to our study. The limited sample size did not allow us to evaluate sex differences. It is possible that raloxifene affects neural activation related to emotion processing differentially in male and female patients. In addition, while the crossover design has the strength of comparing the same person in different conditions, the use of a crossover design can also be a limitation due to the potential for any effects of treatment in the first period carrying over into the second placebo period for those who received raloxifene first. In that instance, we would be less likely to detect changes associated with raloxifene; however, the carryover effect did not appear to negatively influence our results given the effects of raloxifene on brain activity reported. Although raloxifene has a relatively short half-life, raloxifene may have prolonged effects on the brain cells; therefore, future studies should use a parallel group design to assess possible long-lasting effects. In addition, our sample was not large enough to separate out individuals taking different antipsychotic medications; a larger study would allow determination of whether raloxifene may have different effects in the presence of particular antipsychotics. Our sample consisted of patients displaying mild-to-moderate symptom severity on the positive and negative syndrome scale, and it is possible that raloxifene may have a more beneficial effect on symptoms in patients with more severe symptomatology. Last, we were unable to obtain some task behavioral data for several participants due to technical problems, which may have impacted our ability to detect whether raloxifene had a statistically significant effect on emotional face recognition accuracy. However, others^97, 98, 99, 100^ have demonstrated that BOLD activity can be a more sensitive measure of change than measures of behavior and can predict behavioral decline. These studies illustrate the ability of fMRI to reveal significant neurobiological effects between groups in the absence of behavioral differences, which may explain our findings of activation differences in the ROIs while performance measures did not vary.

Notwithstanding the limitations, we believe our study provides the first evidence of significant effects of raloxifene treatment on neural activation during a key component of social processing. Owing to the clinical importance of social impairment in schizophrenia and its relationship to poor functional outcomes, the development of new treatments to improve this core deficit is one of the most pressing challenges in the field. Our findings of increased activity in the hippocampus and IFG during the recognition of emotional faces with raloxifene suggest that adjunctive raloxifene treatment facilitates hippocampal and IFG activity in men and women with schizophrenia. These findings indicate that future research on the use of raloxifene to treat social impairment in schizophrenia is warranted. Given the emerging evidence regarding the efficacy of psychosocial treatments such as cognitive behavioral therapy and social skills training for improving social functioning in schizophrenia,^101, 102^ optimal outcomes may arise from a combination of pharmacologic therapy such as raloxifene with behavioral interventions.

In conclusion, our double-blind, randomized, placebo-controlled, crossover trial found that raloxifene treatment at 120 mg per day enhanced brain activation in key regions involved in facial emotion recognition deficits in schizophrenia and thus, there is potential for using adjunctive raloxifene treatment to reverse social impairment in schizophrenia.

## Figures and Tables

**Table 1:** Demographics, clinical characteristics and blood analyses of patients (*n*=20)

*Variable*	*Baseline*	*Placebo treatment*	*Raloxifene treatment*	*df*	t*/*Z *value*	P
Age (years)	36.5 (8.5)					
Education (years)	13.4 (2.1)					
						
*WAIS-III FSIQ*						
Estimated full-scale IQ	92.4 (10.0)					
						
*WTAR*						
Estimated premorbid IQ	104.0 (6.5)					
						
Sex (M/F)	12/8					

*Race (total)*						
Caucasian	15					
Asian	1					
Caucasian/Asian	3					
Other	1					
						
Handedness (right/left)	19/1					
						
*Diagnosis*						
Schizophrenia	14					
Schizoaffective	6					
						
Age of onset	23.7 (6.3)					
Illness duration	12.9 (6.8)					
						
*Number of hospitalizations*						
0–5	13					
>5	7					
						
Antipsychotic dose (CPZ equivalent)	703.0 (590.0)	699.0 (592.0)	680.7 (574.7)	19	1.34	0.18
% On prolactin-raising antipsychotics	55%					
						
*Hormone assays*						
Prolactin (mIU ml^−1^)	619.8 (654.6)	500.3 (457.7)	484.2 (474.4)	19	1.45	0.15
Luteinizing hormone	5.2 (5.0)	6.6 (6.8)	5.1 (4.6)	19	<0.001	1.0
Follicle stimulating hormone	9.2 (23.3)	9.1 (22.9)	9.8 (20.2)	19	2.07	0.04
						
*PANSS*						
Positive	14.5 (4.9)	14.4 (5.7)	13.4 (4.4)	19	2.03	0.06
Negative	14.8 (6.4)	14.5 (4.7)	14.1 (5.7)	19	0.54	0.6
General	30.7 (7.9)	28.4 (6.6)	28.5 (6.9)	19	0.14	0.89
Total	60.0 (16.6)	57.3 (13.9)	56.0 (13.8)	19	0.76	0.46
						
*Second-generation antipsychotics*						
Olanzapine	4					
Clozapine	1					
Amisulpride	2					
Risperidone	2					
Aripiprazole	1					
Ziprasidone	1					
Quetiapine	1					
Clozapine+paliperidone	1					
Clozapine+amisulpride	2					
Clozapine+aripiprazole	1					
Clozapine+chlorpromazine	1					
Risperidone+quetiapine fumerate	1					
						
*Second- and first-generation antipsychotics*						
Clozapine+haloperidol	1					
Zuclopenthixol+olanzipine	1					

Abbreviations: CPZ, chlorpromazine; PANSS, Positive and Negative Syndrome Scale; WAIS-III FSIQ, Wechsler Adult Intelligence Scale third edition Full-Scale Intelligence Quotient; WTAR, Wechsler Test of Adult Reading. Prolactin-raising antipsychotics: amisulpride, chlorpromazine, haloperidol, flupentixol, paliperidone, risperidone, zuclopenthixol. Prolactin-sparing antipsychotics: aripiprazole, asenapine, clozapine, quetiapine fumerate, olanzapine, ziprasidone. Total number of participants receiving any prolactin-raising medication is 11. The other nine participants were receiving one or a combination of prolactin-sparing antipsychotics. Standard deviation in parentheses.

**Table 2:** Whole-brain analysis showing regions of activation during anger processing in people with schizophrenia during raloxifene treatment and placebo condition

*Brain region*	*Brodmann area*	*L/R*	*CS*	*Peak coordinate*			T
				x	y	z	
*Raloxifene treatment: main effect*							
Lingual gyrus (extends to middle occipital and inferior occipital gyrus)	18	R	1321	10	−86	−10	6.30
Lingual gyrus (extends to fusiform gyrus, cerebellum and parahippocampal gyrus)	18	L	1110	−18	−88	−8	6.29
Fusiform gyrus	37	R	166	50	−50	−10	4.46
Inferior frontal gyrus (extends to middle frontal gyrus and insula)	44	L	2900	−44	24	24	7.66
Inferior frontal gyrus (extends to insula)	47	R	119	30	34	6	4.82
Middle temporal gyrus	19	R	48	36	−82	16	4.31
Middle occipital gyrus	19	L	104	−32	−84	16	6.33
Middle frontal gyrus	46	R	373	50	28	24	4.65
Inferior parietal lobe	7	L	242	−32	−48	44	5.37
Medial frontal gyrus (extends to medial and superior frontal gyrus, limbic lobe and cingulate gyrus)	8	L	475	−8	16	50	5.99
							
*Placebo: main effect*							
Middle frontal gyrus (extends to inferior frontal gyrus)	46	L	535	−48	28	24	6.77
Middle frontal gyrus (extends to inferior frontal gyrus)	9	R	81	42	12	32	4.68
Middle frontal gyrus	6	L	32	−50	8	46	4.59
Precentral gyrus	6	L	42	−42	−4	44	5.47
Medial frontal gyrus (extends to superior frontal gyrus)	6	R	222	4	14	52	5.66

Abbreviations: CS, cluster size (voxels); L, left hemisphere; R, right hemisphere; *T*, *t*-value.

Data are Montreal Neurological Institute coordinates for activation significant at false discovery rate, *P*<0.05.

**Table 3:** Region of interest analyses showing significant treatment effects

*Contrast*	*L/R*	*CS*	x	y	z	T	Z	*Brain region*
Raloxifene > placebo	L	86	−44	−48	4	5.80^a^	4.35	Inferior frontal gyrus
	R	36	34	−14	−24	4.71^a^	3.79	Hippocampus
								
Placebo > raloxifene	None							

Abbreviations: CS, cluster size; L, left hemisphere; R, right hemisphere; *T*, *t*-value; *Z*, *z*-value.

a Small volume family-wise error corrected, *P*<0.05.

Significant activation differences in the hippocampus and inferior frontal gyrus following raloxifene treatment compared with placebo. Centers of activation clusters are given by Montreal Neurological Institute stereotactic coordinates (*x*, *y*, *z*).
