# Supplementary figures and images for: Adjunctive selective estrogen receptor modulator increases neural activity in the hippocampus and inferior frontal gyrus during emotional face recognition in schizophrenia
Source: Transl Psychiatry. 2016 May 3;6(5):e795–. doi: 10.1038/tp.2016.59 (PMC5070055; doi:10.1038/tp.2016.59)

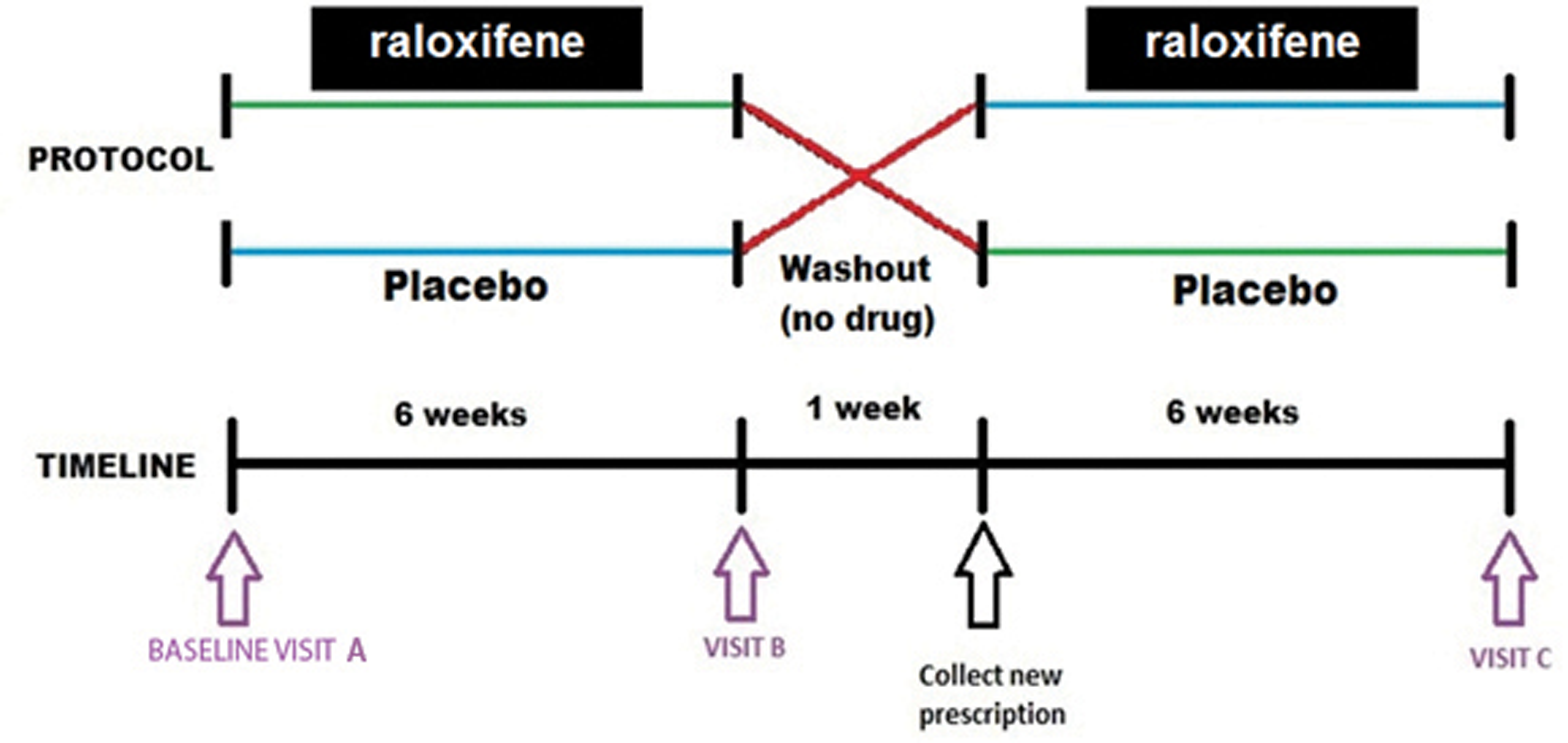

Supplement: Supplementary Figure 1 [file tp201659x1.tif]

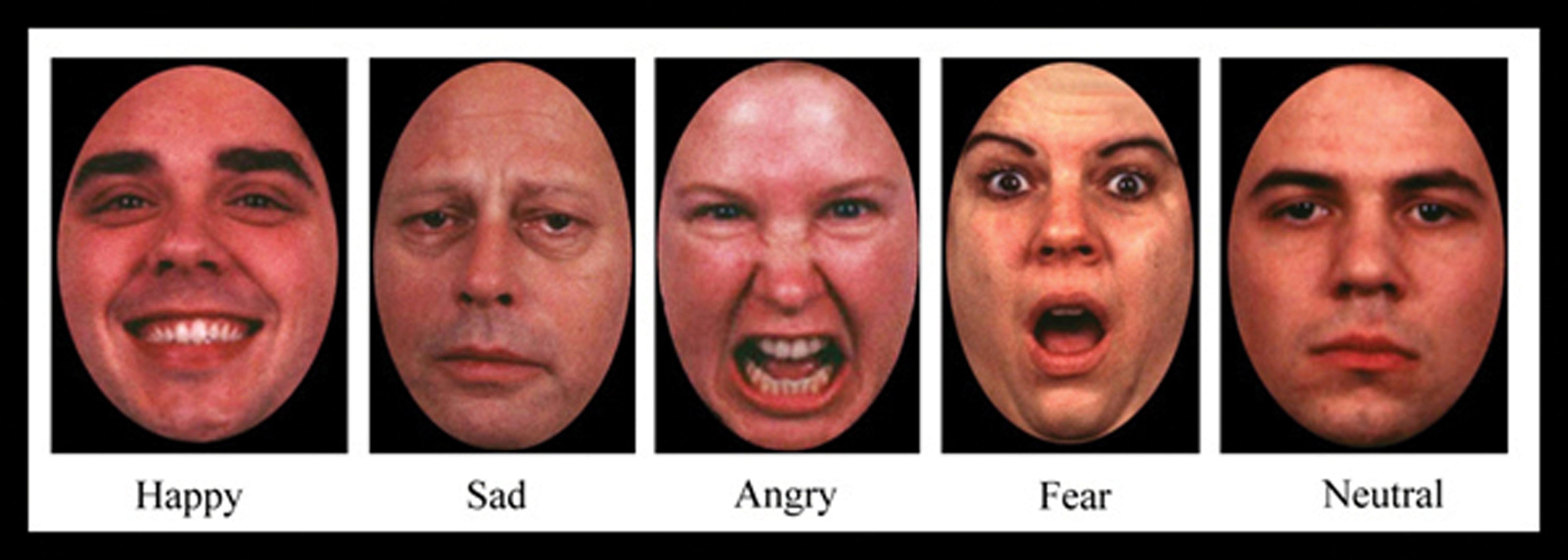

Supplement: Supplementary Figure 1 [file tp201659x2.tif]
