# Supplementary material for: Adjunctive selective estrogen receptor modulator increases neural activity in the hippocampus and inferior frontal gyrus during emotional face recognition in schizophrenia
Source: Transl Psychiatry. 2016 May 3;6(5):e795–. doi: 10.1038/tp.2016.59 (PMC5070055; doi:10.1038/tp.2016.59)
Supplement: Supplementary Information [file tp201659x3.doc]

**Supplementary Information**

**Supplementary Figure 1.** Trial design. Outcome measures for crossover analyses were assessed at week 6 and 13.

**Supplementary Figure 2.** Facial expression depicting various emotions. The present study only assessed brain activation for angry and neutral face recognition.

**Supplementary Table 1.** Mean reaction times (milliseconds) and performance accuracy (% correct) for people with schizophrenia during raloxifene treatment and placebo condition (SD in parentheses).

File format of all Supplementary Figures is JPG (CMYK mode).
