# Supplementary material for: Adjunctive selective estrogen receptor modulator increases neural activity in the hippocampus and inferior frontal gyrus during emotional face recognition in schizophrenia
Source: Transl Psychiatry. 2016 May 3;6(5):e795–. doi: 10.1038/tp.2016.59 (PMC5070055; doi:10.1038/tp.2016.59)
Supplement: Supplementary Table [file tp201659x4.doc]

|  | **Raloxifene** | **Placebo** | **Z** | **p** |
| --- | --- | --- | --- | --- |
| **Angry RT** | 2624 (460) | 2537 (489) | 1.48 | .14 |
| **Angry Accuracy** | 87% (10%) | 90% (10%) | .62 | .53 |
| **Neutral RT** | 2502 (651) | 2342 (511) | .82 | .41 |
| **Neutral Accuracy** | 84% (17%) | 80% (17%) | .90 | .93 |
